# Sub-10 nm Ta Channel Responsible for Superior Performance of a HfO_2_ Memristor

**DOI:** 10.1038/srep28525

**Published:** 2016-06-23

**Authors:** Hao Jiang, Lili Han, Peng Lin, Zhongrui Wang, Moon Hyung Jang, Qing Wu, Mark Barnell, J. Joshua Yang, Huolin L. Xin, Qiangfei Xia

**Affiliations:** 1 Nanodevices and Integrated Systems Laboratory, Department of Electrical and Computer Engineering, University of Massachusetts, Amherst, MA 01003, USA; 2 Center for Functional Nanomaterials, Brookhaven National Laboratory, Upton, NY 11973, USA; 3 Ionic and Electronic Device and Materials Laboratory, Department of Electrical and Computer Engineering, University of Massachusetts, Amherst, MA 01003, USA; 4 Air Force Research Laboratory, Information Directorate, Rome, NY 13441, USA

## Abstract

Memristive devices are promising candidates for the next generation non-volatile memory and neuromorphic computing. It has been widely accepted that the motion of oxygen anions leads to the resistance changes for valence-change-memory (VCM) type of materials. Only very recently it was speculated that metal cations could also play an important role, but no direct physical characterizations have been reported yet. Here we report a Ta/HfO_2_/Pt memristor with fast switching speed, record high endurance (120 billion cycles) and reliable retention. We programmed the device to 24 discrete resistance levels, and also demonstrated over a million (2^20^) epochs of potentiation and depression, suggesting that our devices can be used for both multi-level non-volatile memory and neuromorphic computing applications. More importantly, we directly observed a sub-10 nm Ta-rich and O-deficient conduction channel within the HfO_2_ layer that is responsible for the switching. This work deepens our understanding of the resistance switching mechanism behind oxide-based memristive devices and paves the way for further device performance optimization for a broad spectrum of applications.

Memristive^1^,^2^, or also called resistance switching devices have attracted extensive interest as promising candidates for non-volatile memory^3^, reconfigurable switches^4^, bio-inspired neuromorphic computing^5^ and radiofrequency switches^6^. Albeit with simple metal/insulator/metal (MIM) structures, memristive devices have shown highly desirable properties including low power consumption^7^, fast switching speed^8^, great scalability^9^ and cycling ability^10^,^11^. To date, a wide variety of material systems have been developed for memristive devices that work under different mechanisms. For electrochemical metallization memory (ECM) systems^12^, it has been observed that formation and dissolution of metallic filaments (e.g., Cu or Ag) are responsible for the low and high resistance states^13^,^14^. On the other hand, for valence change memory (VCM) type of systems such as Pt/TiO_2_/Pt/Ti^15^ and Ta/TaO_x_/Pt^11^, it is widely accepted that the motion of oxygen anions (or equivalently the positive-charged oxygen vacancies) leads to valence changes of the metal (cations) and hence the resistance changes of the metal oxide materials. The conduction channel could be a newly formed conductive crystalline sub-oxide phase such as Ti_4_O_7_ in TiO_2_ based devices^16^,^17^ or an amorphous metal-oxygen solid solution such as Ta(O) in TaO_x_ systems^18^. Recently, based on scanning tunneling microscopy (STM) studies it was proposed that the migration of cations, in addition to oxygen anions, could also contribute to the resistive switching behavior in typical VCM materials such as TaO_x_, TiO_x_ and HfO_x_^19^. However, direct visual observations of conduction channels induced by cations migration inside the switching oxides and a physical model concerning the roles of both cations and anions during the resistance switching in VCM-type devices have yet to be revealed.

We herein report a Ta/HfO_2_/Pt memristor that has low programming voltage, fast switching speed (≤5 ns), record high endurance (1.2 × 10^11^ cycles), and reliable retention (extrapolated to be ≫10 years at 85 °C). We also programmed the device to 24 resistance levels with long retention by controlling the compliance currents (CCs). Furthermore, we demonstrated over a million (2^20^) potentiation and depression epochs with this device using electrical pulse trains. Using scanning transmission electron microscopy (STEM) and electron energy loss spectroscopy (EELS), we directly identified a Ta-rich and O-deficient conduction channel that is responsible for the resistance switching. Finally, we proposed a physical model and attributed the switching behavior to the composition modulation of a sub-10 nm conduction channel implemented through the motion of both cations and anions in the oxide layer driven by electric field and thermal effect.

## Results

### Electrical performance characterization

**Figure 1:**
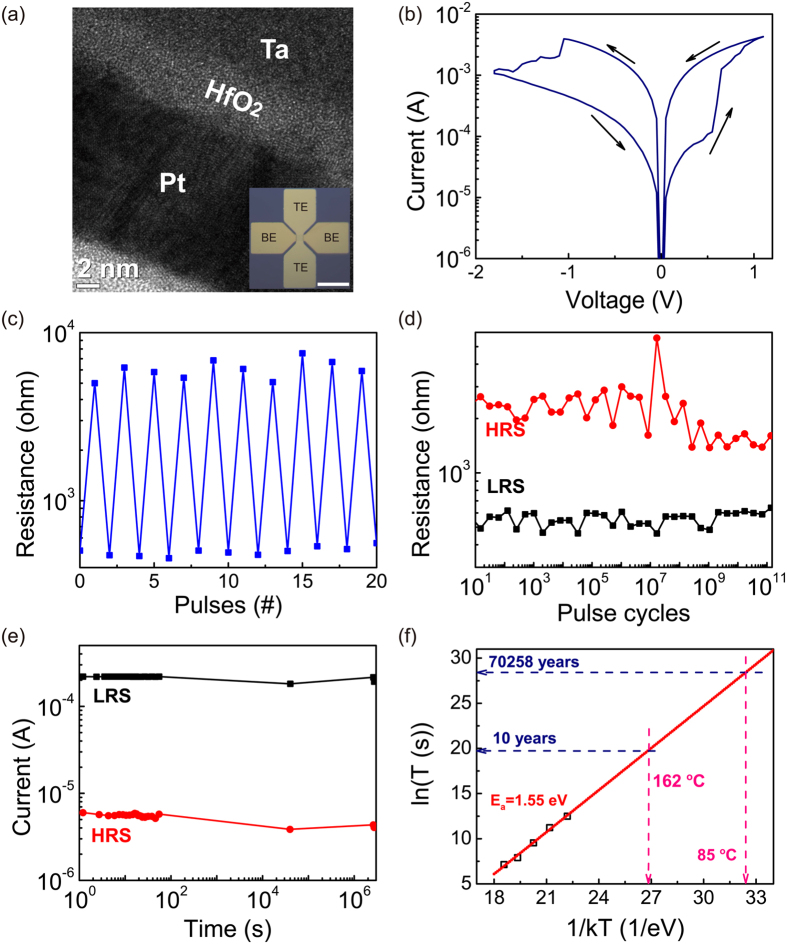
Geometry and electrical performance of the Ta/HfO_2_/Pt cross point memristor. (**a**) TEM cross-section view of the Ta/HfO_2_/Pt device. The inset shows an optical top-view image of the device (scale bar: 50 μm). During all the measurements, the top Ta electrodes were biased while the bottom Pt electrodes were grounded. (**b**) A typical I-V curve from the 10 by 10 μm^2^ crossbar device showing the resistive switching behavior; the black arrows indicate the switching directions. After the forming process, the device stays in ON state. It can then be reset with a negative voltage sweep and then set with a positive voltage sweep. (**c**) The device can be repeatedly switched between HRS and LRS with 5 ns pulses (SET: 2.2 V; RESET: −4 V) indicating faster than 5 ns switching speed. (**d**) 120 billion switching cycles have been demonstrated with pulses of 1.3 V/100 ns for SET and −3.05 V/100 ns for RESET. (**e**) Retention test at room temperature shows no evident degradation after 1 month. (**f** ) Retention time measured at 250, 275, 300, 325 and 350 °C. The dotted red line is the Arrhenius fitting which yields an extrapolated activation energy 1.55 eV. The extrapolated retention time at 85 °C is 70258 years, and is 10 years at 162 °C.

Our cross point device consisted of a 20 nm thick Pt bottom electrode (BE), a 5 nm thick HfO_2_ and a 50 nm thick Ta top electrode (TE) (see *Methods* for fabrication details). HfO_2_ was chosen as the switching layer because it is widely used as the gate dielectric in metal oxide semiconductor field effect transistors. Figure 1a is a typical cross sectional high-resolution TEM (HRTEM) image of the device, which clearly shows both the top and bottom metal/oxide interfaces. The inset of Fig. 1a shows a top view optical image of the device. After electroforming at around 2.02 V (Figure S1 in the Supplementary Information), the 10 × 10 μm^2^ device was brought to low resistance state (LRS). Since then the device exhibited repeatable resistance switching behavior with a negative voltage sweep on the TE resetting it to a high resistance state (HRS) and a positive voltage sweep setting it to a LRS again. The typical Vset and Vreset in the quasi-DC sweeps were about 0.65 and −1.10 V, respectively (Fig. 1b), and they followed a narrow normal distribution as shown by 50 consecutive cycles (Figure S2).

The device can be switched reliably between the HRS and LRS states using 5 ns electrical pulses of 2.2 and −4 V pulse amplitude for SET and RESET, respectively. The resistance read at 0.1 V DC voltage after each switching event is plotted in Fig. 1c. The pulse switching results demonstrated that our HfO_2_ device can be reversibly switched within 5 ns. It needs to be pointed out that the switching speed measurement was limited by the equipment rather than the device itself. A faster speed could potentially be achieved with an increased pulse amplitude if a shorter pulse were available and a special device structure was designed to allow for the transmission of higher frequency signal to the junction area. For endurance measurement, we used 100 ns pulses (Fig. 1d, Vset = 1.3 V, Vreset = −3.05 V, read at 0.1 V) and recorded over 1.2 × 10^11^ open-loop switching cycles for the device without any feedback or power-limiting circuits. To the best of our knowledge, this is the highest reported endurance for a memristive device with a single layer of oxide as the switching material. It is worth noting that although the device stuck at LRS after the endurance test, it could still be RESET using a negative DC voltage with a larger amplitude (−3.2 V), suggesting even better endurance is possible if a verify-write algorithm were adopted^20^.

The Ta/HfO_2_/Pt device also showed excellent retention properties (Fig. 1e). It retained their resistance states (both LRS and HRS) after over one month (>2.7 × 10^6^ s) without noticeable degradation at room temperature. To further evaluate the retention properties, temperature dependent measurements were carried out for the devices. Figure 1f plots the device HRS failure time (t) as a function of temperature (T), which was 2.7 × 10^5^, 7.5 × 10^4^, 1.4 × 10^4^, 2.7 × 10^3^, and 1.3 × 10^3^  s at 250, 275, 300, 325, and 350 °C, respectively (measurement data shown in Figure S3). The relation was well fitted by the Arrhenius equation 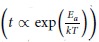, where E_a_ is the activation energy of mobile species and k the Boltzmann constant. The extrapolated retention time at 85 °C was 7 × 10^4^ years, and it was beyond 10 years even at 162 °C, suggesting that our Ta/HfO_2_/Pt devices are promising for non-volatile memory and data storage applications. Interestingly, we observed that the device has a much reliable LRS retention than that of HRS. For example, the device abruptly changed from HRS to LRS after 1.4 × 10^4^ s at 300 °C, while stayed at LRS without evident degradation even after 3 × 10^4^ s (Figure S3c). As a result, we focused on testing the retention properties of the devices at HRS. The longer retention time for the LRS was possibly due to the fact that the conduction filament is relatively strong at LRS^21^,^22^.

The impressive retention performance is fairly surprising given that previously reported retention results for HfO_x_ based devices (using TiN, Hf or Ti as electrodes) are relatively poor^23^,^24^,^25^. The extracted E_a_ from Fig. 1f is 1.55 eV, which is very close to the reported values such as 1.47 eV in Pt/Ta_2_O_5−x_/TaO_2−x_/Pt^10^, and 1.52 eV in Ta/HfO_2_/TiN^23^. On the other hand, great retention property was reported in TaO_x_ devices^26^,^27^. This is an important clue to the working mechanism of our device, which will be discussed in detail later in the mechanism section.

**Figure 2:**
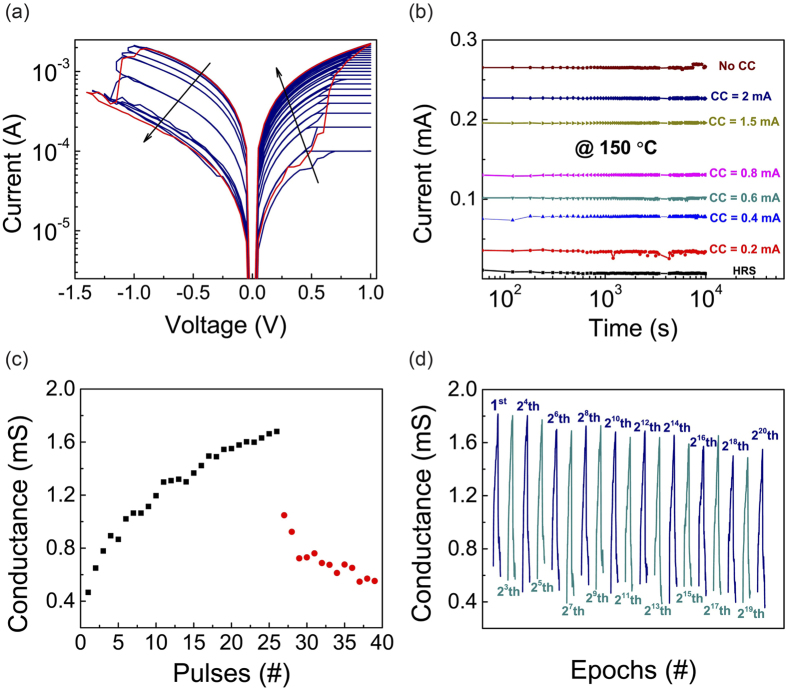
Multilevel non-volatile resistive switching behavior and reliable potentiation/ depression. (**a**) The device can be set by increasing the compliance current and it can be reset with negative voltage sweeps of increased amplitudes to different resistance levels. The red curve shows the IV characteristic without compliance current, and the blue curves indicate switching at different compliances. (**b**) Retention tests of 8 different levels at 150 °C (>10^4^ s), confirming nonvolatile behavior and indicating the device is suitable for multi-level memory. (**c**) A typical analog switching cycle in which the conductance can be gradually increased with 26 positive pulses (100 ns, 0.75 to 1 V, 10 mV step) (potentiation), and gradually decreased with 13 negative pulses (100 ns, −1.05 to −1.17 V, 10 mV step) (depression). (**d**) 2^20^ potentiation/depression epochs, each consists of 39 pulses, have been achieved, suggesting that the device is also promising as electronic synapse for neuromorphic computing.

Multiple resistance states have been achieved for the device by using different compliance currents and stop voltages during programming. As shown in Fig. 2a, the device was set to 24 resistance levels with DC sweeps (0 to 1 to 0 V) by controlling the CCs starting from 100 μA to 3 mA. A higher compliance current led to a lower resistance for the device, which is consistent with previous observation that higher CCs contributed to the continuous growth of conduction channels^28^. On the other hand, the device could be reset to different intermediate resistance levels with negative voltage sweeps of different stop voltages from −1.05 V with a step size of 0.05 V. Retention tests at 150 °C for the same device at 8 different resistance states (including the original HRS and 7 states that were achieved by using different CCs) showed that each state was stable even after >10^4^ s, confirming the nonvolatile behavior and good retention properties. Precise tuning of the resistance into even more states could be implemented through a circuit with one transistor-one memristor (1T1M) configuration^28^, suggesting the potential of our device for the application in multilevel non-volatile memories.

In addition to the multiple discrete levels, the resistance state of the device can be tuned continuously using a train of electrical pulses, in a fashion that is similar to the potentiation and depression of biological synapses^29^. As expected, the application of positive pulses on the TE incrementally increases the device conductance, and the application of negative pulses gradually decreases the conductance. Figure 2c plots the conductance change of the device in response to 39 electric pulses of 100 ns. The amplitudes of the 26 consecutive positive pulses increased from 0.75 to 1 V, and those for the 13 consecutive negative voltages increased from −1.05 to −1.17 V, all with a 10 mV step size. Additionally, we tested the cycling property of this analog conductance modulation behavior in the Ta/HfO_2_/Pt device, and over 2^20^ (>1 million) potentiation/depression epochs have been demonstrated (Fig. 2d). The reliable potentiation and depression behavior indicates that our device is a promising candidate as electronic synapse for neuromorphic computing^5^.

### Switching mechanism study

**Figure 3:**
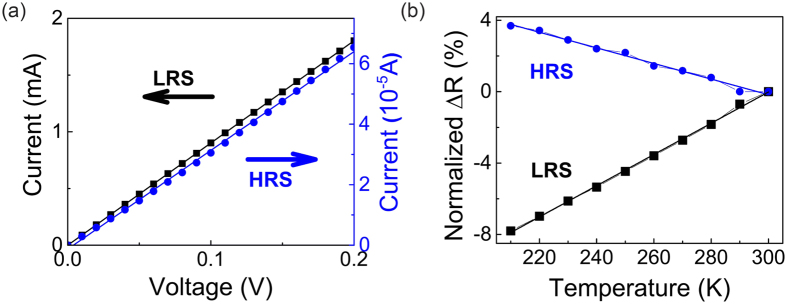
Electrical conduction mechanisms of the LRS and HRS. (**a**) The linear fitting of the I-V curves for LRS and HRS indicates the absence of a tunneling gap between electrode and conduction channel(s). (**b**) Temperature dependence of LRS and HRS resistances from the device. The resistance change (∆R) is normalized by (*R* −* R*(300 *K*))/R(300 *K*). The LRS resistance decreases linearly, while HRS resistance increases linearly with ambient temperature, suggesting a transition of the conduction channel from metallic to non-metallic materials. The TCR is measured to be 8.75 × 10^−4^/K for LRS and −4.37 × 10^−4^/K for HRS. The switching should be attributed to the composition modulation of the conduction channel(s).

We believe the switching of our Ta/HfO_2_/Pt device is due to the composition modulation of a localized conduction channel(s) under electrical and thermal effects. As shown in Fig. 3a, both LRS and HRS have almost linear I-V relation when read at low voltages, indicating the absence of a tunneling gap between the electrode and conduction channel. As such, the switching cannot be attributed to the modulation of tunneling gap size as found in TiO_x_ based devices^30^,^31^. Furthermore, temperature-dependent conduction measurements were performed for both LRS and HRS states (Fig. 3b). In the LRS, the junction resistance increased linearly with temperature, a typical behavior for metallic materials. On the other hand, the HRS resistance changed in an opposite way and decreased with temperature, indicating a non-metallic conduction behavior. The temperature coefficient of resistance (TCR) was calculated to be 8.75 × 10^−4^/K for LRS and −4.37 × 10^−4^/K for HRS. The different signs of the TCR at LRS and HRS suggest the switching was caused by the modulation of the conduction channel composition while not the channel size (TCR is independent of geometry)^18^. Devices with a M1/HfO_2_/M2 stack is a leading candidate for memory applications and thus one of the most extensively used stack, where M1 is a relatively inert metallic layer, such as Pt or TiN, and M2 is a reactive metal layer, such as Hf, Ta and Ti^23^,^32^. Traditionally, those devices have been naturally classified as HfO_2_ devices when mechanisms and performances are discussed. However, the impressive retention observed in our device that is comparable to that for TaO_x_ devices instead of HfO_x_ devices^26^,^27^ implies that it is not a pure HfO_x_ device and the reactive metal electrode plays a more important role than expected in determining the device properties. Specifically, in our device stack, it is highly likely that Tantalum cations have migrated into the HfO_2_ layer and directly contributed to the formation of conduction channel(s).

**Figure 4:**
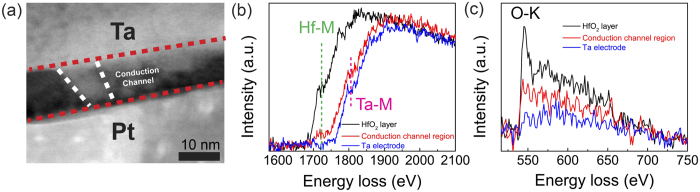
Direct observation of a Ta-rich and O-deficient conduction channel. (**a**) HAADF-STEM image shows a sub-10 nm conduction channel connecting Ta top and Pt bottom electrodes. The conduction channel is brighter in the image, which means it contains more atoms with large atomic numbers (Ta in this case). (**b**) Comparison of core-loss EELS spectra collected at the pristine HfO_2_ layer, conduction channel region and Ta electrode. It indicates the conduction channel is Ta-rich. (**c**) O-K edge EELS spectra taken at three areas, which clearly show the conduction channel is also O-deficient.

To verify our hypothesis, scanning transmission electron microscopy (STEM) and electron energy loss spectroscopy (EELS) analyses were conducted for a Ta/HfO_2_/Pt device with a 3 μm diameter Ta via as the top electrode. A thicker (10 nm) HfO_2_ was used to provide sufficient material volume for a better TEM characterization. The device was repeatedly switched for several cycles and left at LRS before being cut using a focus ion beam (FIB) microscope. During the resistance switching cycles, a small part of the device was deformed because of Joule heating and the evolution of compressed oxygen bubble, consistent with previous results^16^. As shown in previous reports, conduction channels are usually surrounding the deformation sites^33^,^34^, thus the deformation site in our device was used to identify the position of conduction channel(s) during FIB cutting. Figure 4a is a typical high-angle annular dark field (HAADF)-STEM image of a conduction channel connecting the Ta TE and Pt BE. The cone-shaped conduction channel has a diameter of 10 nm at the Ta end and 6 nm near the Pt BE. In addition, some incomplete Ta-rich channels were also found in the same device that did not reach to the Pt bottom electrode (Figure S4, and also shown in the right part of the Fig. 4a). It is noticeable that the conduction channel is brighter than the surrounding oxide, which means it is composed of heavier atoms (i.e., Ta in the device) because the image intensity in the STEM mode is proportional to Z^1.7^ (Z is the atomic number of the species) and the atomic density. Typical core-loss EELS spectrum taken at the unchanged HfO_2_ layer has a dominant Hf-M edge peak at (1716 eV) while that from Ta electrode has a typical Ta-M edge peak at (1797 eV) (Fig. 4b). The EELS spectrum from the conduction channel shows an evident peak of Ta-M edge while only a small bump near Hf-M edge, indicating that the conduction channel is Ta-rich. This observation agrees well with the intensity contrast of the HAADF-STEM image (Fig. 4a). Similarly, O-K edge EELS spectra from those three positions clearly reveal the decreased oxygen intensity within the conduction channel (Fig. 4c). Core-loss EELS mapping results further confirmed the conduction channel is Ta-rich and O-deficient (Figure S5). The contribution of Ta migration to the formation of conduction channels is in line with recent STM studies^19^, and it is believed that the migration of Ta cations is caused by the electric field and Joule heating. The Ta migration may also be responsible for the usually high activation barrier energy observed in some similar device stack, such as 1.52 eV in Ta/HfO_2_/TiN^23^.

Based on the electrical measurements and physical characterization, the switching mechanism of the Ta/HfO_2_/Pt device can be explained as follows. During the forming step, Ta is oxidized into Ta^x+^ when a positive voltage is applied on it. Due to the strong electric field and concentration gradient, the mobile Ta^x+^ cations migrate into the HfO_2_ layer and serve as dopants. In the meantime, O^2−^ anions are attracted towards the Ta TE, which introduces O vacancies (V_O_s) as dopants in the oxide layer. This is similar to the well-studied Ta anodizing process, in which case the tantalum oxide growth on the surface is attributed to both the inward migration of O anions to the metal/oxide interface and the outward migration of Ta cations to the oxide/solution interface^19^,^35^,^36^,^37^. The simultaneous movement of both ions is attributed to their comparable mobility and hence similar migration barrier (0.047 eV difference between Ta cations and O anions) within the HfO_2_^35^,^36^. The continuous migration of Ta^x+^ into and O^2−^ out of the HfO_2_ layer increases the doping levels of Ta and V_O_ to the HfO_2_ layer and finally form localized conduction channel(s), bringing the device to LRS. In the first RESET process, a negative bias is applied at the Ta TE. In the vertical direction (perpendicular to the electrode/oxide interfaces), electric field drives O anions toward Pt BE while pulls Ta cations back toward Ta TE. The motion of both ions leads to lower Ta but higher oxygen concentrations in the conduction channel, and hence a more resistive device that is at HRS. On the other hand, a positive voltage during the first SET process reverses the process and turns the device back to LRS. It is worth noting that although it is easier to understand the channel composition modulation through the electric field induced vertical drift, thermally enhanced lateral diffusion also plays an important role for the device operation^18^. As a result, we attribute the resistive switching in our Ta/HfO_2_/Pt device to the growth and reoxidation of Ta-rich and O-deficient conduction channel(s) through motions of Ta cations and O anions. This theory is very different from previously popular switching mechanism for HfO_2_ based devices, in which only O anions at the electrode/oxide interface contributes to the switching while the electrodes (e.g., Ta, Ti, and Hf) only serve as the oxygen gettering layer^23^. On the other hand, the Ta-rich conduction channel in our HfO_2_ device leads to similar switching behaviors (such as long retention and high endurance) as TaO_x_-based memristive devices^10^,^18^. The amorphous structure of the conduction channel(s) facilitates ion motion and exchanging during the resistive switching process, contributing to the superior endurance and fast switching speed, while the continuous modulation of the channel composition leads to the multiple resistance states of our device^18^. The competition between drift and diffusion is believed to be responsible for the observed analog switching behavior^38^. The long retention of our device could be attributed to the large diffusion barriers (1.55 eV, Fig. 1f) of the mobile ions. *In-situ* TEM studies on nanoscale vertical devices are desired in the future to uncover the whole dynamic switching process (especially the reset step).

Finally, although we directly observed Ta cations migration, the co-existence of O anion movement makes our device fundamentally different from traditional ECM devices based on motion of Ag or Cu ions. The difference is a result from a number of reasons. First, Ta is more easily oxidized than Ag due to the much stronger negative Gibbs energy for the formation of oxides (Ta_2_O_5_: −760 kJ mol^−1^ and Ag_2_O: −60 kJ mol^−1^)^39^. Consequently, the motion of O anions facilitates the oxidation of Ta and contributes to the modulation of device resistances. On the contrary, metallic filaments of Ag or Cu can stably exist within the oxide layer. Second, there is a big difference in the migration barriers between Ag and O (1.19 eV for Ag^40^ and 1.52 eV for O anions in TaO_x_) while that between Ta and O is very small. As a result, motion of Ag or Cu ions is more preferable than O anions in ECM devices. The transition from VCM- to ECM- type switching behaviors in typical VCM material system (Ta/TaO_x_/Pt) has been achieved by inserting a thin amorphous carbon layer at Ta/TaO_x_ interface^19^ or using highly reduced TaO_x_ to suppress the role of O anions^41^.

## Discussion

We have developed a Ta/HfO_2_/Pt memristive device with fast switching speed (≤5 ns), record high endurance (1.2 × 10^11^ cycles), and reliable retention (extrapolated to be ≫10 years at 85 °C). We also achieved 24 non-volatile long-retention resistance states by controlling compliance current during DC programming, and over a million (2^20^) potentiation and depression epochs using electrical pulse trains. The device performance suggests that our devices can be used for both memory and computing applications. More importantly, we studied the switching mechanism and attributed the switching to the composition modulation of a sub-10 nm Ta-rich O-deficient conduction channel by both anion and cation migration under electric field and thermal effect. The results strongly suggest that the reactive metal electrode in the oxide based memristors plays a much more important role than previously expected in determining the switching mechanism and device performance. We further built a model that successfully explains the excellent behavior of our device. This work broadens our understanding of the resistance switching mechanism behind oxide-based memristive devices and paves the way for further device performance optimization and applications.

## Methods

### Device fabrication

We used Si wafers that have 100 nm thermally grown SiO_2_ on top as the substrates. For the 10 × 10 μm^2^ micro-devices, the bottom electrodes were patterned by ultraviolet photolithography. After that, 1.5 nm Ti/20 nm Pt were deposited sequentially by electron beam evaporator, followed by a lift-off process in acetone. A 5 nm HfO_2_ blanket layer was prepared by atomic layer deposition (ALD) using water and tetrakis(dimethylamido)hafnium as precursors at 250 °C. The 50 nm thick Ta top electrodes were defined by a second photolithography step and a 15 s O_2_ descum, metallization using DC sputtering and liftoff. The devices for FIB cutting were prepared in the similar fashion, except that a blank 20 nm Pt/1.5 nm Ti layer was deposited as the BE, followed by the ALD of 10 nm HfO_2_. A 40 nm thick SiO_2_ was sputtered on as isolation layer and a 3 μm wide via hole was opened using photolithography and reactive ion etching before the 50 nm thick 75 μm wide Ta pad was deposited as the TE.

### Electrical Characterization

The DC electrical characterizations were carried out with an Agilent 4156B semiconductor parameter analyzer in a voltage-sweep mode. Pulse measurements for switching speed, cycling endurance and analog switching were conducted with an Agilent 81160a pulse generator. The devices were programmed to ON or OFF states and the resistance was read at 100  mV DC voltage between switching events. The retention tests at 250, 275 and 300 °C were performed on a Cascade Summit 11000 probe system equipped with a thermal chuck (ambient to 300 °C, 0.1 °C accuracy). The retention performances at 325 and 350 °C were measured in a variable temperature micro probe system (MMR Technology) (70 to 730 K, ± 0.1 K accuracy). The device resistances were periodically monitored by Agilent B1500 at different temperatures in every 60 s with a low read voltage (0.1 V, ~20 ms) to avoid disturbance of the device states. For all the electrical measurements, the bottom electrodes were grounded while the top electrodes were biased.

### Physical Characterization

The HAADF-STEM images, and EELS analysis were acquired in an aberration-corrected Hitachi HD2700C Scanning Transmission Electron Microscope operated at 200 keV. The FIBed TEM cross-section samples were prepared with FEI Helios 600 nanoLab.

## Additional Information

**How to cite this article**: Jiang, H. *et al*. Sub-10 nm Ta Channel Responsible for Superior Performance of a HfO_2_ Memristor. *Sci. Rep.* **6**, 28525; doi: 10.1038/srep28525 (2016).

## Supplementary Material

Supplementary Information
