# Supplementary material for: Sub-10 nm Ta Channel Responsible for Superior Performance of a HfO2 Memristor
Source: Sci Rep. 2016 Jun 23;6:28525. doi: 10.1038/srep28525 (PMC4917839; doi:10.1038/srep28525)
Supplement: Supplementary Information [file srep28525-s1.pdf]

Supplementary Information

Sub-10 nm Ta Channel Responsible for Superior Performance of a HfO_2 Memristor

Hao Jiang[1], Lili Han[1,2], Peng Lin[1], Zhongrui Wang[1,3], Moon Hyung Jang[1,3], Qing Wu[4], Mark Barnell[4], J. Joshua Yang[3], Huolin Xin[2], and Qiangfei Xia[1*]

[1] Nanodevices and Integrated Systems Laboratory, Department of Electrical and Computer Engineering, University of Massachusetts, Amherst, MA 01003, USA.

[2] Center for Functional Nanomaterials, Brookhaven National Laboratory, NY 11973, USA.

[3] Ionic and Electronic Device and Materials Lab, Department of Electrical and Computer Engineering, University of Massachusetts, Amherst, MA 01003, USA.

[4] Air Force Research Laboratory, Information Directorate, Rome, NY 13441, USA.

[*]E-mail: qxia@ecs.umass.edu

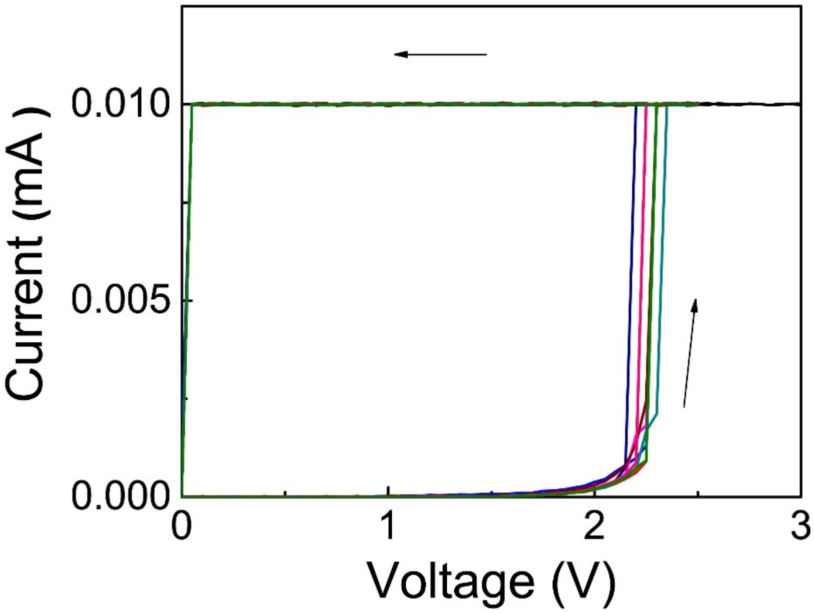

Figure S1. Typical I-V curves for the electroforming process from 10 randomly chosen Ta/HfO_2/Pt devices. The forming voltages are around 2.2 V. After the forming process, the devices stay at ON state.

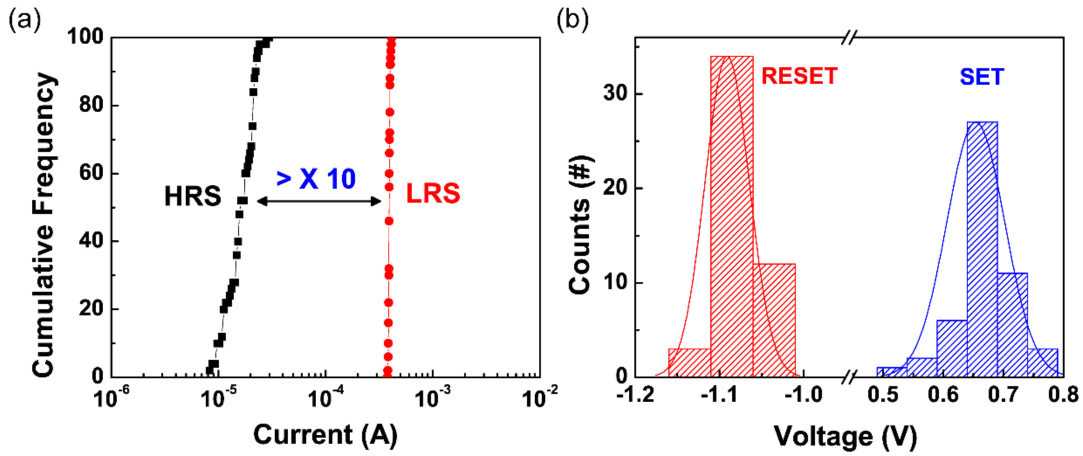

Figure S2. (a) Distributions of both LRS and HRS resistances from 50 consecutive DC sweeps. The window is kept > 10 X. (d) Statistics of SET and RESET voltages, which are normally distributed around 0.65 V and -1.1 V separately. Device size: 10×10 μm^2.

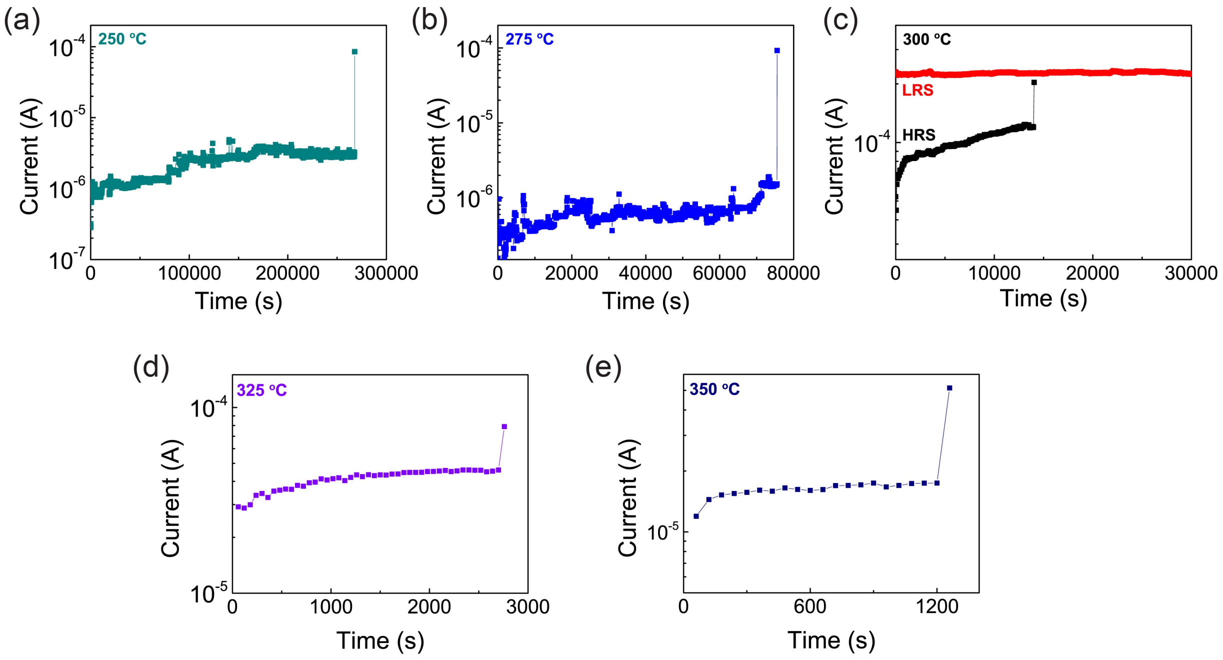

Figure S3. Retention measurements of the device at (a) HRS at 250 °C, (b) HRS at 275 ºC, (c) LRS and HRS at 300 ºC, (d) HRS at 325 °C and (e) HRS at 350 °C. The failure time for the HRS are 2.7×10^5, 7.5×10^4, 1.4×10^4, 2.7×10^3, and 1.3×10^3 s, respectively.

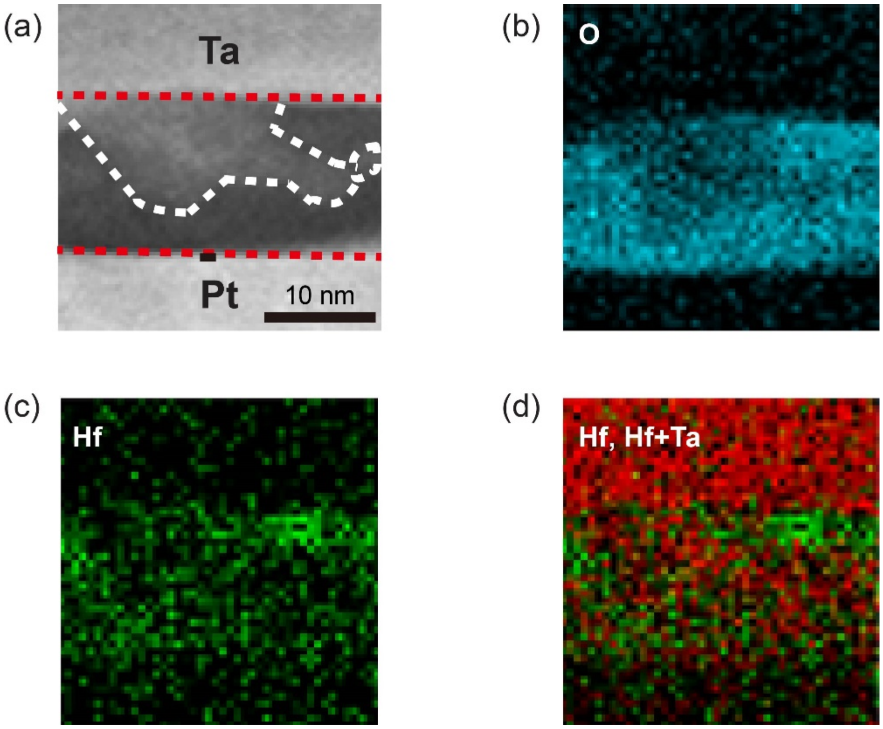

Figure S4. HAADF-STEM image and core-loss EELS mapping of an incomplete conduction channel. (a) HAADF-STEM image of an incomplete conduction channel. The EELS mapping results for (b) O, (c) Hf, (d) combination of Hf and Hf+Ta suggest the incomplete conduction channel is also Ta-rich and O-deficient.

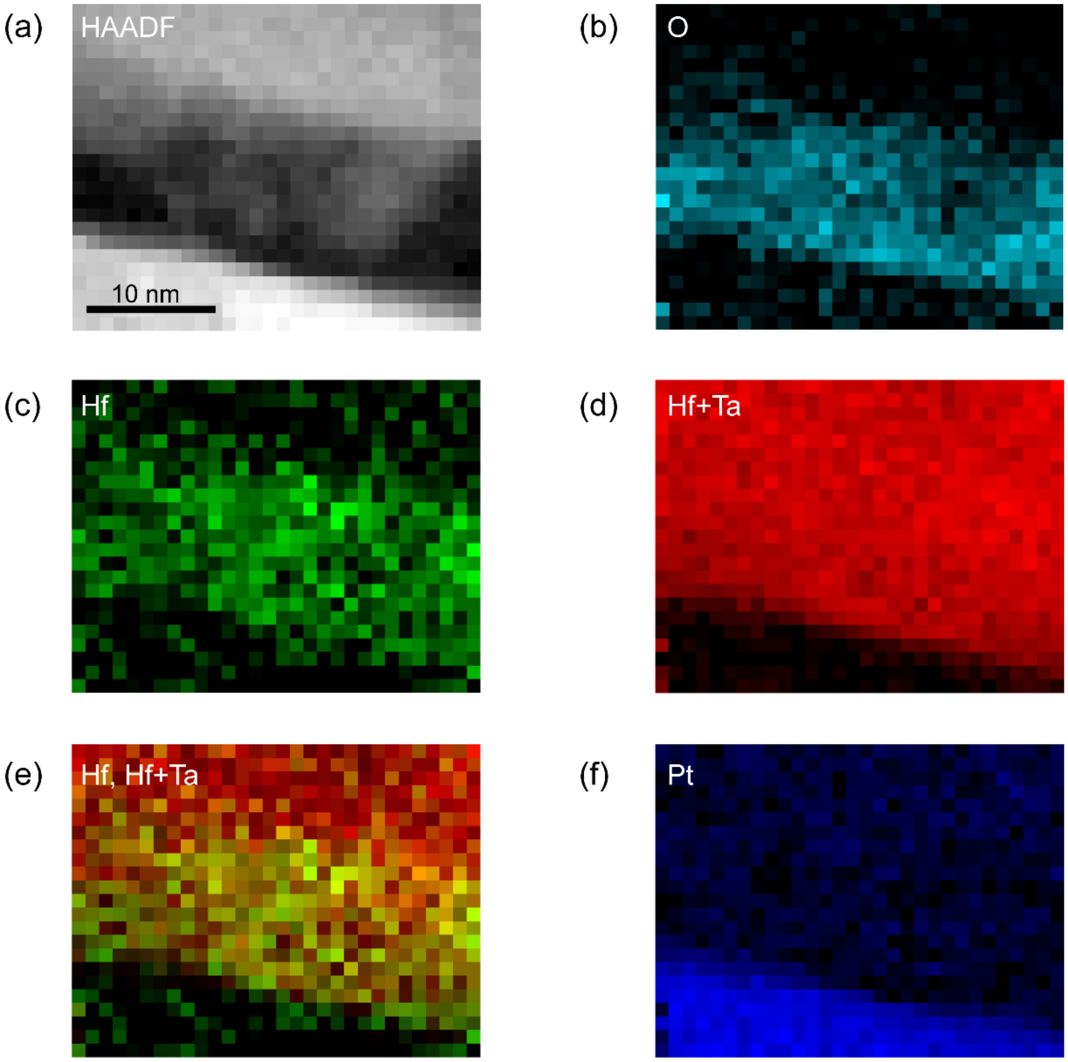

Figure S5. STEM-EELS mapping of the conduction channel area. (a) HAADF-STEM image of the mapped region generated during mapping. (b) The EELS mapping result for O, clearly shows the conduction channel region is oxygen-deficient. The EELS mapping results for (c) Hf, (d) Hf + Ta, (e) combination of Hf and Hf + Ta, and (f) Pt indicate the conduction channel contains more Ta, which is consistent with the brighter intensity of the conduction channel in (a).
